# Concealment of Potential Exposure to COVID-19 and Its Impact on Outbreak Control: Lessons from the HIV Response

**DOI:** 10.4269/ajtmh.20-0449

**Published:** 2020-05-26

**Authors:** Alvin Kuo Jing Teo, Rayner Kay Jin Tan, Kiesha Prem

**Affiliations:** 1 Saw Swee Hock School of Public Health, National University of Singapore, National University Health System, Singapore, Singapore;; 2 Department of Infectious Disease Epidemiology, Faculty of Epidemiology and Population Health, London School of Hygiene and Tropical Medicine, London, United Kingdom

## Abstract

Globally, more than 4 million people have been infected with COVID-19, and more than 300,000 deaths have been reported across 188 countries. Concealment of one’s potential exposure to the virus has negative implications for the spread of COVID-19 across the socio-ecological spectrum, including the futility of contact-tracing efforts, exposure of frontline staff, and the spread of COVID-19 in the community. We draw lessons learned from HIV to discuss stigma and the attribution of blame surrounding the phenomenon of concealment of one’s potential exposure to COVID-19 using a socio-ecological perspective. This article also illustrates the psychosocial aspect of the disease, and the negative repercussions of concealment of potential exposure on transmission in the community and to front-liners, healthcare resources, and outbreak containment.

COVID-19 is now a pandemic, with more than four million people infected and more than 300,000 deaths reported across 188 countries worldwide.^1^ In the early phases of the pandemic, most international borders remained open, and social or cultural events that involved mass gatherings of people were held. That changed quickly as countries began reporting the importation of new infections and, subsequently, the identification of local clusters. Of late, there have been reports of persons deliberately concealing their travel histories and contacts with those who may have been infected with SARS-CoV-2.^2–9^

The reluctance to divulge potential association with high-risk events can have a grave socio-ecological impact on the management and control of COVID-19. At the individual and interpersonal levels, concealment of potential exposure to SARS-CoV-2 could expose people in the community to infection, contributing to community transmission and clusters. Furthermore, individuals may conceal vital information concerning potential COVID-19 exposure because of stigma and fear^10,11^ when they present themselves to healthcare facilities on onset of symptoms. This poses elevated threats of COVID-19 infection among healthcare workers whom they encounter, especially those working in primary care clinics or outpatient departments that are not adequately equipped to manage suspected cases. Concealment may also negatively impact contact-tracing and isolation efforts, and exacerbate already dwindling healthcare resources, as isolation of healthcare workers is required. As the evidence converges toward frequent asymptomatic transmission,^12,13^ cover-up of crucial information in the absence of classic clinical symptoms further exacerbates the spread of SARS-CoV-2 and challenges containment strategies.

Individuals who were unforthcoming with key information have been reported to fuel community transmission in different localities.^14,15^ These reports suggest fear of being associated with certain events that have faced public scrutiny, such as large-scale religious events and travel to regions severely affected by the pandemic. The fear of being associated with a specific stigmatized identity, such as being identified as gay in the case of a COVID-19 cluster in Itaewon, a gay nightlife district in Seoul, South Korea, might have hindered exposed individuals from stepping forward for COVID-19 testing.^14,16^ Some may conceal their potential exposure to COVID-19 because of the fear of disclosing one’s illegal or stigmatized activities, such as participating as a provider of sexualized labor.^17^ Failures in providing complete and timely information, and delays in seeking appropriate care, including testing, could lead to further transmission of SARS-CoV-2 in the community.

Everyone is affected by the pandemic, but some people are at higher risk of infection because of being on the front line of the response against COVID-19. As COVID-19 continues to spread, healthcare workforces and systems are strained by the rising number of new infections, hospitalizations, and deaths. Since the beginning of the pandemic, more than 3,000 health workers have been infected in China, and a significant proportion of frontline health workers in Italy have been infected and succumbed to COVID-19.^2^ Healthcare workers are susceptible to exposure and infection through encounters with people who have a subclinical infection, and this risk is exacerbated by long working hours, the lack of protective equipment, and, more recently reported, encounters with people who deliberately hid critical information about potential exposure to SARS-CoV-2.^5–9^ In instances in which healthcare workers become infected, they may transmit SARS-CoV-2 to other vulnerable populations in healthcare facilities. On identification, exposed front-liners would have to be tested and quarantined, further compounding the already dire healthcare workforce crunch.

As countries begin to ease lockdown measures, fear lingers, especially in places that were greatly affected by the first wave of the pandemic. Performing acts that might be perceived as socially irresponsible will reinforce stigma and lead to a vicious cycle of concealment, delayed testing and diagnosis, and a rebound of the outbreak. From the population health perspective, concealment of potential exposure to COVID-19 will lead to inaccurate information, making contact tracing for epidemiological links impossible. Without effective contact tracing, it will be challenging to roll out containment measures such as quarantine of people who might have contracted COVID-19. With a rise in propagation of infections in the community, a curve that has been flattened may peak and exert increased pressure on the healthcare system.

This phenomenon of concealment of one’s stigmatized health status or the circumstances that surround its acquisition may sound all too familiar to researchers, clinicians, and policymakers in the fields of HIV and TB science, where stigma and the attribution of blame to individuals have been key barriers to achieving optimal health outcomes for patients and those who care for them. Although we struggle to deal with such issues in the context of COVID-19, we already have the answers through our experience with the HIV response that should allow us to act swiftly and effectively. Through our experience with HIV, we know that individuals may conceal their HIV status out of fear of potential social judgment.^18^ Attribution theory, which has been a prominent theory to explain how and why individuals stigmatize others based on perceptions of control around their disease acquisition,^18^ may also provide insight into the underpinnings of stigmatizing attitudes. As such, although it might not be possible or practical to conceal one’s status in the context of COVID-19, concealing one’s participation in activities that may have placed an individual at risk of SARS-CoV-2 acquisition may occur to avoid stigma and discrimination that arise from such forms of attribution.^18^

Health workers need to be protected to maintain resilient health systems and safeguard continuous patient care. Similarly, community transmissions have to be kept low to prevent cases from overwhelming healthcare facilities and to prevent vulnerable populations from experiencing severe health outcomes. From the policy and legal perspective, enacting relevant laws and regulations could deter people from concealing vital information. However, this presupposes a robust contact-tracing system to detect and ascertain past contacts, over and above self-reported contacts from COVID-19 patients. Thus, criminalization or enforcement laws to deter nondisclosure of contacts may be counterproductive. Instead, to obtain truthful accounts of past exposures, a more pragmatic approach that we can learn from the HIV response would be to place less focus on stigmatizing the ways in which people acquired the virus and rather address the stigma that accompanies such admissions. This can be enacted at the institutional level when contact tracing and declarations take place. Learning from the success of anonymous HIV testing and its effect on testing uptake and linkage to medical care,^19^ stigmas associated with COVID-19 can potentially be alleviated to encourage people to come forward for testing using the same approach.^20^ That said, more can be done upstream to prevent such forms of exposure in the first place. It is thus vital not to overlook the importance of public education and to raise awareness on social responsibility that is contextualized and embedded in the value frameworks of those who may be at risk for acquiring SARS-CoV-2. An understanding of why individuals prioritize activities at the expense of transmission risk would allow us to develop community-based interventions to bridge the gaps that exist between what we deem as socially responsible behaviors and the culturally informed behaviors that people engage in.

The race is on to find effective treatment and vaccines for COVID-19. Drawing experiences from the HIV pandemic and other contemporary epidemics, we must recognize that the psychosocial aspect of the disease is imminent in outbreak control, and its importance should be acknowledged in tandem with other biomedical approaches. The concealment of potential exposure stemming from fear and stigma has negative repercussions on communities and front-liners. As countries fight to contain outbreaks, a holistic approach is needed to curb transmission. Current policies and institutional strategies mostly involve authorities proactively finding, isolating, and treating people who contracted COVID-19. To further contain the infection, everyone has a role to play and that includes practicing good personal hygiene, social responsibility, and an enabling environment for people who might be at risk of SARS-CoV-2 to provide honest accounts of their past exposure and to step forward for testing if warranted.
